# Current management of intracerebral haemorrhage in China: a national, multi-centre, hospital register study

**DOI:** 10.1186/1471-2377-11-16

**Published:** 2011-01-30

**Authors:** Jade W Wei, Yining Huang, Ji-Guang Wang, Ming Liu, Lawrence KS Wong, Qifang Huang, Li'e Wu, Emma L Heeley, Hisatomi Arima, Craig S Anderson

**Affiliations:** 1 The George Institute for Global Health, Royal Prince Alfred Hospital and University of Sydney, Sydney, Australia; 2 Department of Neurology, Peking University First Hospital, Beijing, China; 3 Centre for Epidemiological Studies and Clinical Trials, Ruijin Hospital, Shanghai Jiaotong University School of Medicine, Shanghai, China; 4 Department of Neurology, West China Hospital, Sichuan University, Chengdu, China; 5 Prince of Wales Hospital, Chinese University of Hong Kong, Hong Kong, China; 6 The First Hospital Affiliated to the Baotou Medical College, Baotou, China

## Abstract

**Background:**

We aimed to examine current practice of the management and secondary prevention of intracerebral haemorrhage (ICH) in China where the disease is more common than in Western populations.

**Methods:**

Data on baseline characteristics, management in-hospital and post-stroke, and outcome of ICH patients are from the ChinaQUEST (QUality Evaluation of Stroke Care and Treatment) study, a multi-centre, prospective, 62 hospital registry in China during 2006-07.

**Results:**

Nearly all ICH patients (n = 1572) received an intravenous haemodiluting agent such as mannitol (96%) or a neuroprotectant (72%), and there was high use of intravenous traditional Chinese medicine (TCM) (42%). Neurosurgery was undertaken in 137 (9%) patients; being overweight, having a low Glasgow Coma Scale (GCS) score on admission, and Total Anterior Circulation Syndrome (TACS) clinical pattern on admission, were the only baseline factors associated with this intervention in multivariate analyses. Neurosurgery was associated with nearly three times higher risk of death/disability at 3 months post-stroke (odd ratio [OR] 2.60, p < 0.001). Continuation of antihypertensives in-hospital and at 3 and 12 months post-stroke was reported in 732/935 (78%), 775/935 (83%), and 752/935 (80%) living patients with hypertension, respectively.

**Conclusions:**

The management of ICH in China is characterised by high rates of use of intravenous haemodiluting agents, neuroprotectants, and TCM, and of antihypertensives for secondary prevention. The controversial efficacy of these therapies, coupled with the current lack of treatments of proven benefit, is a call for action for more outcomes based research in ICH.

## Background

Intracerebral haemorrhage (ICH) is the most serious and least treatable form of stroke, with up to 50% of patients dying and half of survivors left with significant disability [1]. Despite considerable research effort in recent decades, there is still no convincing evidence of benefit from any medical treatment, the role of surgery remains controversial, and the chances of surviving ICH has failed to improve in developed countries over recent decades [1,2]. Although ICH occurs in about 10-15% of all strokes in Western populations, it has high impact in terms of acute and long-term medical care costs as well as productivity loss, estimated to be US$6 billion annually in the United States alone [3]. In China, as in other parts of Asia, ICH accounts for up to a third of all strokes [4], which, taken together with the very large at-risk population, makes ICH a much more common disease in this region. Yet, there are few published studies of the management of ICH in China, and thus limited knowledge of how patterns of treatment more often reflect local customs and practice rather than guideline recommendations. Such data can serve the basis for planning interventions to improve outcomes. In this study, we examined the current management of ICH in China, with a focus on medical intervention both on the acute phase and in the uptake of antihypertensive therapy for secondary prevention.

## Methods

The ChinaQUEST (QUality Evaluation of Stroke Care and Treatment) study used a prospective, multi-centre, hospital-based, registry design to collect data on patients admitted to hospital with acute stroke across a wide range of urban sites in mainland China over a 5-month period in 2006. The study methods and objectives have been described in detail elsewhere [5,6]. A network of 62 hospitals selected from each of the Chinese government's 8 defined economic regions participated in the study, with the following characteristics: 53 (85%) with teaching status; 46 (74%) with >500 beds; 40 (64%) with >500 stroke admissions per annum; 60 (97%) with neurosurgical services; and neurology bed numbers ranged from 16 to 160 (average 62 beds). Key neurology investigators at each participating hospital were asked to recruit 50-300 consecutive stroke patients (age ≥15 years) except those with subarachnoid haemorrhage who would agree to 4 interview-based assessments at: baseline (after admission), hospital discharge, and 3 and 12 months follow-up. Written informed consent was obtained from all patients, or an appropriate family member (in situations where the patient was disabled) to participate. De-identified data were transferred to electronic Case Record Forms on a secure website connected to a central, password-protected, database located at The George Institute for Global Health in Sydney, Australia. The study was approved by the ethics committees of Peking University First Hospital (Beijing), Ruijin Hospital (Shanghai), Prince of Wales Hospital (Hong Kong), and The University of Sydney. Good Clinical Practice guidelines in accordance with the Declaration of Helsinki were used and the privacy of patients was strictly protected.

Information on baseline sociodemographic situation was obtained predominantly by face-to-face interviews, in-hospital details including diagnosis of stroke subtype were obtained through medical records and interviews with patients or proxies, and follow-up details were obtained primarily through telephone interviews. Baseline information collected included patient socio-demographic characteristics, clinical features, pathological stroke type, and history of co-morbid cardiovascular (CV) risk factors. Diagnostic and management approaches employed in-hospital (including neuroimaging, intravenous/oral medications, supportive care, and surgery), medication use at 3 and 12 months post-stroke, and various outcome measures of patients on discharge and upon follow-up, were also recorded. Specific outcomes recorded included in-hospital complications (pneumonia, deep venous thrombosis, recurrent stroke, urinary tract infection, other sepsis, pulmonary embolus, coronary event, seizure, fall with injury, or any other clinically significant event that prolonged hospital stay), modified Rankin Scale [mRS] score, death, and discharge destination.

Baseline characteristics considered in the analyses included sociodemographic variables, medical history, clinical and hospital features. Sociodemographic variables taken into account consisted of: age, sex, living alone, low education (primary education only or illiterate), and main lifetime occupation type. Medical history variables investigated included: high CV risk (defined as having ≥2 of the following self-reported co-morbidities diagnosed pre-stroke: history of hypertension, diabetes, hyperlipidaemia, atrial fibrillation, prior stroke, prior transient ischaemic attack, or prior coronary artery disease [includes prior heart attack or angina]), current cigarette smoking, regular alcohol consumption within the 3 months prior to stroke onset and being overweight (body mass index [BMI] ≥24 [7]). Clinical features considered included time from symptom onset to hospital presentation, 'poor' Glasgow Coma Scale (GCS) score on admission (defined as 3-8 of a top score of 15), and the Oxfordshire Community Stroke Project (OCSP) clinical classification of stroke [8,9]. Hospital features investigated here included level, size, teaching status, and location as either in a 'rich' or 'poor' provincial area classified according to whether the 2006 Gross Regional Product per capita for the province was above or below the national average (2006 Gross Domestic Product per capita 16 084 CNY [10] [US $2298, based on an exchange rate of US $1 being equivalent to 7 CNY, which is used throughout]). Hospitals were also geographically defined as being either 'northern' (includes the areas of Beijing, Hebei, Heilongjiang, Henan, Inner Mongolia, Jilin, Liaoning, Qinghai, Shaanxi, Shandong, Tianjing, and Xinjiang) or 'southern' (includes the areas of Anhui, Chongqing, Fujian, Guangdong, Guangxi, Hong Kong, Hubei, Hunan, Jiangsu, Jiangxi, Shanghai, Sichuan and Yunnan province).

Logistic regression analysis was used to determine factors associated with any form of neurosurgical intervention (includes open craniotomy haematoma evacuation, shunt insertion, and microsurgery haematoma evacuation) of ICH. Univariate analyses were first conducted, followed by multivariate modelling adjusting for age, sex and significantly different (p < 0.05) baseline variables. In all models examined, hospital was also introduced as a random effect to account for any clustering at the hospital level. Multivariate logistic regression analysis was also used to determine the effect of surgery on outcome, adjusting for all potential confounders including age, sex, marital status, living alone, low level of education, active employment (part time or full time), annual household income, prior dependency, high CV risk based on self-reported and post-stroke diagnoses, current cigarette smoking, regular alcohol consumption, being overweight, time of presentation to hospital, poor admission GCS, OCSP classification; in-hospital use of intravenous steroid, neuroprotectant, hemodiluting agents and traditional Chinese medicine (TCM); and antihypertensive use during the 3 months post-stroke. Data are reported with odds ratios (OR) and 95% confidence intervals (CI). All analyses were conducted using STATA 11.1 (StataCorp LP, College Station, TX).

## Results

In total, 13038 patients were screened for eligibility, but 6530 (50%) were excluded by investigators as the final diagnosis was not stroke or stroke of recent onset (30.9%), declined to participate (16.1%), rapid death (1.5%), or other reasons (1.5%). A total of 6508 patients were enrolled but 154 were excluded from analyses due to uncertainty of diagnosis and missing data. Thus, we included 6354 stroke cases of which 1572 (25%) were due to ICH which were confirmed by computerised tomography (CT) or magnetic resonance imaging (MRI) with the exception of one case.

Table 1 shows that the ICH patients tended to be middle aged, have a history of hypertension, and to present within 6 hours of symptom onset. Of the various management options, intravenous TCM, various neuroprotectant agents (such as intravenous edaravone, ganglioside GM1, cattle encephalon glycoside and ignotin, cinepazide, citicholine) and haemodiluting agents including mannitol, were all in exceptionally high use (Table 2). Table 3 shows that the most commonly used neuroprotectant was citicholine. Among those patients with specified neuroprotectant use (n = 809), the majority (92.8%) used a single neuroprotectant, with 4 being the maximum number of neuroprotectants used in any 1 patient. In-hospital mortality was 13% and more than half of survivors were reported being disabled/dependent at the time of hospital discharge.

**Table 1:** Baseline characteristics of ICH patients*

	All N = 1572
*Sociodemographic*	
Age, mean (SD), years	61.1 ± 12.9
Female	632 (40)
Living alone	77 (5)
Low education (primary only or illiterate)	820 (52)
Main lifetime occupation type	
Manual work^†^	850 (54)
None reported	162 (10)
	
*Medical history*	
History of hypertension	1033 (66)
History of diabetes mellitus	122 (8)
History of hyperlipidaemia	143 (9)
Prior stroke	355 (23)
Prior coronary artery disease^‡^	150 (10)
Current smoker	399 (25)
Regular alcohol consumption^§^	411 (26)
Overweight^||^	637 (41)
	
*Medication history (within one month of stroke onset)*	
Antihypertensive	730 (46)
Antiplatelet	215 (14)
Warfarin	2 (0.1)
Lipid lowering	36 (2)
Hypoglycaemic	88 (6)
	
*Clinical features*	
Time from symptom onset to hospital presentation	
Median (IQR), hours	3.5 (1.3,17.0)
<6 hours	922 (59)
Unknown	29 (2)
Transported to hospital by ambulance	663 (42)
Poor GCS score on admission^#^	360 (23)
Clinical classification - TACS**	290 (18)

*Values are reported as mean ± SD, median (IQR), or number (percentage) of subjects

^†^Manual work includes construction, farming/forestry/fishing and related, installation and related, manufacture and production, transportation and driver occupations

^‡^Coronary artery disease includes prior heart attack or angina

^§^Within the 3 months prior to stroke onset

^||^Defined as body mass index ≥24; percentage based on non-missing values, 1 missing value overall

^#^GCS - Glasgow Coma Scale, poor score ≤8 in range 3 (low) to 15 (high, normal); percentage based on non-missing values, 64 missing values overall

**Oxfordshire Community Stroke Project Clinical Classification where TACS is Total Anterior Cerebral Syndrome

**Table 2:** Management and outcome of ICH patients in-hospital*

Investigations/treatment/outcome at discharge	All N = 1572
*Investigations*	
Computerised tomography	1552 (99)
Magnetic resonance imaging	112 (7)
	
*Supportive care*	
Allied health therapist^†^	320 (20)
Traditional Chinese medicine therapist	121 (8)
Assisted feeding	326 (21)
	
*Medical treatment*	
Antihypertensive therapy	1007 (64)
Intravenous traditional Chinese medicine	657 (42)
Intravenous neuroprotectant	1130 (72)
Intravenous haemodiluting agents (eg mannitol)	1511 (96)
Intravenous corticosteroids	158 (10)
In neurology ward with stroke unit	380 (24)
Neurosurgical intervention	
Open craniotomy haematoma evacuation	59 (4)
Shunt insertion	2 (0.1)
Microsurgery haematoma evacuation	59 (4)
Other	17 (1)
	
*Outcome at discharge*	
Experienced ≥1 in-hospital complication^‡^	378 (24)
Length of hospital stay, median (IQR)	18.0 (9.0,26.0)
Outcome (as measured by modified Rankin Scale)	
0-2	560 (36)
3-5	802 (59)
6	201 (13)
Discharge destination for survivors	
Home	1227 (78)
Alternate hospital	110 (7)
Other^§^	34 (2)

*Values are reported as median (IQR) or number (percentage) of subjects

^†^Allied health therapist includes speech & language therapist, physiotherapist, occupational therapist, rehabilitation professional, and social worker

^‡^Complications include pneumonia, deep venous thrombosis, recurrent stroke, urinary tract infection, other sepsis, pulmonary embolus, coronary event, seizure, fall with injury, or any other clinically significant event that prolonged hospital stay

^§^Includes family/friends home and nursing home/institution and unknown

**Table 3:** Characteristics of neuroprotectant use

Neuroprotectant	n (%*)
Edaravone	95 (8.4)
Ganglioside GM1	29 (2.6)
Cattle encephalon glycoside and ignotin	112 (9.9)
Cinepazide	56 (5.0)
Citicholine	334 (29.6)
Other^†^	198 (17.5)
Unspecified	321 (28.4)

*As a proportion of all patients on any neuroprotectant therapy (n = 1130); groups are not mutually exclusive as 1 patient may be on more than 1 neuroprotectant

^†^Use of agents including deproteinised calf blood extracts, adenosine triphosphate, cerebroprotein extracts, cytidine disodium triphosphate, lysine, piracetam, meclofenoxate, and sodium fructose diphosphate.

Overall, neurosurgical intervention was undertaken in 137 (9%) patients, with as many patients receiving open craniotomy as CT-guided microcatheter evacuation of the haematoma of ICH. Logistic regression analysis examining the relationship between neurosurgery and baseline variables and outcome (Table 4) shows that being overweight, having a poor GCS score on admission, and a large hemispheric (TACS) syndromic pattern to the ICH, were the only baseline factors associated with neurosurgical management in multivariate analyses. Neurosurgery was associated with increased risk of death/disability at 3 months post-stroke (OR 2.60; 95% CI 1.55, 4.35) after adjusting for known potential confounders.

**Table 4:** Logistic regression analysis of factors associated with neurosurgical management of ICH

Surgical intervention	Odds ratio (95% CI)	
	
	Crude n = 1572*	Adjusted n = 1555
*Socio-demographic factors*		
Age	0.98 (0.96, 0.99)	0.98 (0.96, 0.99)
Female	0.88 (0.60, 1.30)	0.84 (0.56, 1.27)
Living alone	1.49 (0.68, 3.24)	
Low education (primary only or illiterate)	1.07 (0.72, 1.59)	
Main lifetime occupation type^†^		
Manual work	1.01 (0.66, 1.55)	
None reported	0.94 (0.48, 1.82)	
		
*Hospital factors*		
Level three vs level two category	2.77 (0.87, 8.85)	
Large size (>500 beds)	1.63 (0.53, 5.00)	
Teaching status	2.02 (0.49, 8.38)	
Situated in a rich provincial area	1.11 (0.42, 2.90)	
Situated in northern China	0.97 (0.37, 2.53)	
		
*Medical history*		
≥ 2 cardiovascular risk factors^‡^	0.70 (0.46, 1.07)	
Current smoker	1.14 (0.74, 1.76)	
Regular alcohol consumption	1.37 (0.91, 2.07)	
Overweight (body mass index ≥24)	1.72 (1.17, 2.52)	1.68 (1.12, 2.51)
		
*Clinical features*		
Time from symptom onset to hospital presentation		
<6 hours	1.41 (0.93, 2.11)	
Unknown	-^§^	
Poor admission GCS score	4.04 (2.70, 6.04)	3.83 (2.51, 5.83)
Clinical classification - TACS	3.08 (1.84, 5.16)	2.23 (1.30, 3.83)

*Model with overweight was run on n = 1571 excluding 1 missing value, and model with severe admission GCS score was run on n = 1556 excluding 16 missing values

^†^Reference group: non-manual work including management, professional and related, service, sales/commercial, armed forces, and clerical/administration support

^‡^Defined as ≥2 of the following: history of hypertension, diabetes, hyperlipidaemia, atrial fibrillation; prior stroke, prior transient ischaemic attack, and prior coronary artery disease

^§^Model cannot be run, no observations in outcome group for those whose time from symptom onset to hospital presentation is unknown

Uptake of antihypertensive use in-hospital was evaluated in 1104 surviving patients with complete data at all follow-up time points. The frequency of antihypertensive therapy in-hospital and at 3 and 12 months post-stroke was 71%, 74% and 72%, respectively, in all patients (n = 1104); and 78%, 83%, 80%, respectively, in patients with 'hypertension' (n = 935) either diagnosed prior to or after admission to hospital. In patients who had received in-hospital antihypertensive treatment, the main reasons for discontinuation at 12 months post-stroke were: 'patient had no indication for therapy' or 'patient was not hypertensive' (38%), 'patient did not refill prescription' (34%), and 'patient refused therapy' (18%). Inability to pay was not reported by any patient as a reason for therapy discontinuation.

## Discussion

This study indicates that the management of ICH in China is characterised by high rates of use of proven antihypertensive therapy for secondary prevention but also of high rates of non-evidence based medical therapies, in particular of intravenous haemodiluting agents, neuroprotectants and TCM in the acute phase. Although TCMs (herbal/complementary therapies) have a long tradition of therapeutic benefit for a wide variety of conditions in China, and similarly for the popularity of neuroprotectives in the targeting brain function, they are not recommended in current national stroke guidelines [7]. Nevertheless, doctors trained through the Western medical system in China are also exposed to TCM teachings for 10-15% of their education programme [11]. Thus, most doctors have some knowledge regarding the fundamentals of TCM use and the traditional prescriptions which typically consist of multiple herbs, minerals and/or animal products boiled together to make a drink. However, there is increasing availability and subsequent use of commercially produced compound TCMs in solid or injectable dosage forms, the uses of which are not taught in medical school. Moreover, there appears to be an intrinsic belief that TCMs are effective in the medical profession, even in acute stroke, with 75% of doctors reporting that Chinese herbal products are 'definitely beneficial' in a survey of more than 1000 doctors from 247 hospitals across China during 1993-1994 [12]. Further, while there is still inconclusive evidence regarding the benefit of routine use of haemodiluting agents such as mannitol [13], both national Chinese stroke guidelines and international based guidelines (such as the latest American Heart Association/American Stroke Association guidelines) advocate restricting their use to patients with clear evidence of increased intracranial pressure [7,14]. Whether or not such extensive use of these therapies in China can be attributable to higher rates of increased intracranial pressure associated with ICH or that the medical profession also believes there are additional pleiotropic benefits from mannitol, is unclear. It is important to note, though, that such non-evidence based practice also exists in the West, with the common use of therapies such as prophylactic anticonvulsants which also have a limited evidence base.

In regard to surgical management, those ICH patients with more severe strokes at presentation, as evident by poor GCS scores or TACS pattern, were more likely to be surgical candidates, as has been shown by others [15]. However, why overweight individuals were also more likely to receive surgery is less readily explained. Overall, nearly 1 in 10 (9%) patients received some form of neurosurgical intervention, which is consistent with the 13% reported in a Japanese registry [16], and near the lower end of the spectrum of rates reported internationally that range from 2% in Hungary to 74% in Lithuania [15]. The high uptake of CT-guided microcatheter evacuation of ICH is noteworthy, since it is a promising technique that can be undertaken in low resource settings without the need for neurosurgical expertise [15]. In our study, neurosurgery was associated with increased likelihood of poor outcome after adjusting for potential confounders including initial severity, pre-morbid dependency, CV comorbidities and medications used in the acute and post-acute phase. These findings differ from current evidence indicating beneficial effects of surgery [17] and likely reflect the intervention being undertaken in more severe cases of ICH in our study. Other factors that affect the choice and outcome from surgery include the size and location of the underlying haematoma, which were not recorded in this study.

Although the duration of hospital stay (median 18 days) for ICH patients in our study was comparatively longer than has been documented in other countries such as the United States, Brazil and Israel with mean length of stays of 8-12 days [18-20], it was still much shorter than in Japan, where a 38 day median length of stay for ICH has been reported [21]. Similarly, our finding of 13% in-hospital mortality was lower than 27% in the United States, 28% in Indonesia, and 28% in Israel [19,20,22], but higher than 10% in Japan [21]. This is consistent with ICH being overall a milder disease in Asians compared to non-Asians [1], but there are likely to be cultural differences in management such as those involving withdrawal of life support, rehabilitation, and home care, that are also relevant to influencing both in-hospital and long-term survival. Indeed, Christensen *et al *have shown that sites in China and Singapore that participated in the Factor Seven for Acute Hemorrhagic Stroke (FAST) trial had patients enrolled with lower median GCS scores at the time of randomisation and better 90 day survival compared with patients from sites in other countries [23]. Although comparisons are complicated by methodological differences and temporal variations in case mix and background care, the frequency of hypertension (66%) in our study was comparable to that seen (74%) among patients recruited within 6 hours of ICH into the pilot phase of Intensive Blood Pressure Reduction in Acute Cerebral Haemorrhage Trial (INTERACT), a multi-national clinical trial whose study population consisted mainly of Chinese participants; the higher frequency observed in INTERACT likely attributable to the inclusion criteria of having a high blood pressure level on presentation, and the low frequency of diabetes (8%) found in our study population was also analogous to those in INTERACT (8% overall) [24].

With regards to secondary prevention of ICH, the lowering of blood pressure is the most important element, as in the primary prevention of ICH, and is recommended in both local and international guidelines [7,14]. In this study, approximately 80% of those with a history of hypertension (either self-reported or diagnosed in hospital) were taking antihypertensive treatment, and reassuringly this figure remained near stable over the 12 months of follow-up. For the small proportion of patients who discontinued therapy, the majority of the reasons were patient related either through passive non filling of prescriptions or the active refusal of therapy. Although cost is a potential barrier to continuation of therapy in this setting, an inability to pay was not reported by any patient as a reason for discontinuation. This may be attributable to the inclusion of wealthier patients who could afford hospitalisation and thus subsequent medications in this study, or the widespread availability of inexpensive locally manufactured antihypertensive agents such as nifedipine, nitrendipine, hydrochlorothiazide, metoprolol, captopril, and enalapril [25]. Thus, patient education programmes are warranted to ensure therapy compliance and improved patient outcomes.

We recognise that our study has several limitations. To begin with, only consenting patients who were admitted to participating hospitals with Departments of Neurology were included. As these hospitals included interested neurologist investigators based in generally large well-resourced hospitals located principally in major urban areas, the patterns of management that are described herein may not be representative of what is occurring in smaller and/or rural based hospitals. Nonetheless, given the likely low resources available in such settings, it is likely that any such bias in our study is towards an overestimate of the patterns of care overall. Another limitation is that specific clinical details, such as the location, volume and mass effect of the haematoma in ICH, were not recorded in our study, so that the various factors influencing the decision to undertake neurosurgical intervention was not fully appreciated. Nevertheless, variables related to stroke severity are to some extent captured by the GCS scoring and TACS classification systems. Although we did not use a validated stroke severity scale such as the National Institutes of Health Stroke Scale (NIHSS) due to its inconsistent use in routine care, the much simpler and widely known GCS has been shown to correlate well with the NIHSS score (p < 0.001; correlation coefficient = -0.730) [26]. Similarly, we elected to use the OCSP syndromic classification system for both ischaemic stroke and ICH to avoid logistical complexities over gathering reliable information on haematoma topography to categorise ICH. We recognise, though, that the OCSP system has not been validated in ICH, raising the potential for misclassification bias. However, given that all investigators were given the guidelines for the OCSP classification system which they likely used in association with findings from neuroimaging to enter the data, it is likely that we achieved a tolerably useful classification of ICH, albeit by location into 'posterior' (brainstem/cerebellum) versus 'anterior' (hemisphere), and of the severity (TACS versus PACS/LACS) of the haemorrhage/haematoma.

Another factor to acknowledge is the difficulty of assessing the agreement between the management strategies employed in the acute phase of ICH and those specified by local guidelines, given the lack of data available on factors that would guide use of these interventions. Moreover, our measures of baseline medical history were prone to recall bias, and the results are subject to survivor bias as a small proportion of the patients with severe disease are likely to have died before enrolment. However, self-reported medical history have been shown to correlate well with medical records [27], and any investigation of current management is likely to be most beneficial to those who survive the first few hours after the onset of ICH, thus the effect of these issues on the results are likely to be minimal. Furthermore, the consistency of our findings to those in neighbouring Japan provides some reassurance of the validity of our data.

## Conclusions

In summary, this study documents current acute management and uptake of secondary prevention for ICH in China. With its massive population undergoing rapid aging and other demographic transitions, China faces an increasingly heavy burden of stroke across a variety of health care settings. Despite encouraging high rates of secondary preventative antihypertensive use in this urban hospital network, there is still much room for improvement with patient/physician education campaigns. Furthermore, given the lack of therapies of proven benefit in ICH and the high use of therapies of controversial efficacy, there is clear need for evaluations to improve ICH outcomes and reduce the current heavy reliance on non-evidence based practices in the acute setting.

## Competing interests

The authors declare that they have no competing interests.

## Authors' contributions

JWW designed the study, prepared and analysed the data, interpreted the results and drafted the manuscript. YH, JGW, ML, LKSW, QH, LW, and ELH participated in the acquisition of data and critical revision of the manuscript. ELH and HA advised on methods of data analysis, presentation of results, and critical revision of the manuscript. YH, JGW and CSA conceived the study. CSA contributed to design of the study, advised on interpretation and presentation of results, and participated in critical revision of the manuscript. All authors read and approved the manuscript.

## Pre-publication history

The pre-publication history for this paper can be accessed here:

http://www.biomedcentral.com/1471-2377/11/16/prepub

## References

[B1] van Asch CJ Luitse MJ Rinkel GJ van der Tweel I Algra A Klijn CJ Incidence, case fatality, and functional outcome of intracerebral haemorrhage over time, according to age, sex, and ethnic origin: a systematic review and meta-analysis Lancet Neurol 2010 9 167 176 10.1016/S1474-4422(09)70340-0 20056489

[B2] Adeoye O Woo D Haverbusch M Sekar P Moomaw CJ Broderick J Flaherty ML Surgical management and case-fatality rates of intracerebral hemorrhage in 1988 and 2005 Neurosurgery 2008 63 1113 1117 discussion 1117-1118 10.1227/01.NEU.0000330414.56390.DE 19057323 PMC2717618

[B3] Rincon F Mayer SA Clinical review: Critical care management of spontaneous intracerebral hemorrhage Crit Care 2008 12 237 10.1186/cc7092 19108704 PMC2646334

[B4] Zhang LF Yang J Hong Z Yuan GG Zhou BF Zhao LC Huang YN Chen J Wu YF Proportion of different subtypes of stroke in China Stroke 2003 34 2091 2096 10.1161/01.STR.0000087149.42294.8C 12907817

[B5] Heeley EL Anderson CS Huang Y Jan S Li Y Liu M Sun J Xu E Wu Y Yang Q Role of health insurance in averting economic hardship in families after acute stroke in China Stroke 2009 40 2149 2156 10.1161/STROKEAHA.108.540054 19359646

[B6] Wei JW Arima H Huang Y Wang J Yang Q Liu Z Liu M Lu C Heeley EL Anderson CS for the ChinaQUEST Investigators Variation in the frequency of intracerebral haemorrhage and ischaemic stroke in China: a national, multicentre, hospital register study Cerebrovasc Dis 2010 29 321 327 10.1159/000278927 20130397

[B7] Chinese Centre for Disease Control, Chinese Medical Association Neurology Division China Guideline for Cerebrovascular Disease Prevention and Treatment 2007 Beijing: People's Medical Publishing House

[B8] Anderson CS Taylor BV Hankey GJ Stewart-Wynne EG Jamrozik KD Validation of a clinical classification for subtypes of acute cerebral infarction J Neurol Neurosurg Psychiatry 1994 57 1173 1179 10.1136/jnnp.57.10.1173 7931376 PMC485481

[B9] Bamford J Sandercock P Dennis M Warlow C Burn J Classification and natural history of clinically identifiable subtypes of cerebral infarction The Lancet 1991 337 1521 1526 10.1016/0140-6736(91)93206-O 1675378

[B10] National Bureau of Statistics of China China Statistical Yearbook 2007 Book China Statistical Yearbook 2007 2007 City: China Statistics Press

[B11] Hesketh T Zhu WX Health in China. Traditional Chinese medicine: one country, two systems BMJ 1997 315 115 117 9240055 10.1136/bmj.315.7100.115 PMC2127090

[B12] Chen Z Sandercock P Xie J-X Peto R Collins R Liu L-S Hospital management of acute ischemic stroke in China Journal of Stroke and Cerebrovascular Diseases 1997 6 361 367 10.1016/S1052-3057(97)80219-4 17895034

[B13] Bereczki D Fekete I Prado GF Liu M Mannitol for acute stroke Cochrane Database Syst Rev 2007 CD001153 17636655 10.1002/14651858.CD001153.pub2 PMC7032636

[B14] Morgenstern LB Hemphill JC Anderson C Becker K Broderick JP Connolly ES Jr Greenberg SM Huang JN MacDonald RL Messe SR Guidelines for the management of spontaneous intracerebral hemorrhage: a guideline for healthcare professionals from the American Heart Association/American Stroke Association Stroke 2010 41 2108 2129 10.1161/STR.0b013e3181ec611b 20651276 PMC4462131

[B15] Gregson BA Mendelow AD International variations in surgical practice for spontaneous intracerebral hemorrhage Stroke 2003 34 2593 2597 10.1161/01.STR.0000097491.82104.F3 14563963

[B16] Morioka J Fujii M Kato S Fujisawa H Akimura T Suzuki M Kobayashi S Surgery for spontaneous intracerebral hemorrhage has greater remedial value than conservative therapy Surgical Neurology 2006 65 67 72 10.1016/j.surneu.2005.03.023 16378863

[B17] Prasad K Mendelow AD Gregson B Surgery for Primary Supratentorial Intracerebral Hematoma. A Meta-Analysis of 10 Randomized Controlled Trials Stroke 200919797178

[B18] Christensen MC Valiente R Sampaio Silva G Lee WC Dutcher S Guimaraes Rocha MS Massaro A Acute treatment costs of stroke in Brazil Neuroepidemiology 2009 32 142 149 10.1159/000184747 19088487

[B19] HCUP HCUPnet. Healthcare Cost and Utilization Project (HCUP): Nationwide Inpatient Sample (NIS) Book HCUPnet. Healthcare Cost and Utilization Project (HCUP): Nationwide Inpatient Sample (NIS) 2006 2006 City: Agency for Healthcare Research and Quality, Rockville, MD 21413206

[B20] Tanne D Goldbourt U Koton S Grossman E Koren-Morag N Green MS Bornstein NM A national survey of acute cerebrovascular disease in Israel: burden, management, outcome and adherence to guidelines Isr Med Assoc J 2006 8 3 7 16450742

[B21] Yoneda Y Okuda S Hamada R Toyota A Gotoh J Watanabe M Okada Y Ikeda K Ibayashi S Hasegawa Y Hospital cost of ischemic stroke and intracerebral hemorrhage in Japanese stroke centers Health Policy 2005 73 202 211 10.1016/j.healthpol.2004.11.016 15978963

[B22] Misbach J Ali W Stroke in Indonesia: a first large prospective hospital-based study of acute stroke in 28 hospitals in Indonesia J Clin Neurosci 2001 8 245 249 10.1054/jocn.1999.0667 11386799

[B23] Christensen MC Broderick J Vincent C Morris S Steiner T Global differences in patient characteristics, case management and outcomes in intracerebral hemorrhage: the Factor Seven for Acute Hemorrhagic Stroke (FAST) trial Cerebrovasc Dis 2009 28 55 64 10.1159/000219298 19468216

[B24] Anderson CS Huang Y Wang JG Arima H Neal B Peng B Heeley E Skulina C Parsons MW Kim JS Intensive blood pressure reduction in acute cerebral haemorrhage trial (INTERACT): a randomised pilot trial Lancet Neurol 2008 7 391 399 10.1016/S1474-4422(08)70069-3 18396107

[B25] Liu L Wang W Yao C for the <<Chinese Hypertension Prevention and Treatment Guidelines>> (Basic edition) committee, Chinese Centre for Disease Control, the National Centre for CVD Control and Research, and the Chinese Hypertension League [2009 Basic edition of the <<Chinese Hypertension Prevention and Treatment Guidelines>> (Excerpts)] Zhongguo Yi Xue Qian Yan Zha Zhi 2010 2 60 74

[B26] Cheung RT Zou LY Use of the original, modified, or new intracerebral hemorrhage score to predict mortality and morbidity after intracerebral hemorrhage Stroke 2003 34 1717 1722 10.1161/01.STR.0000078657.22835.B9 12805488

[B27] Margolis KL Lihong Q Brzyski R Bonds DE Howard BV Kempainen S Simin L Robinson JG Safford MM Tinker LT Phillips LS Validity of diabetes self-reports in the Women's Health Initiative: comparison with medication inventories and fasting glucose measurements Clin Trials 2008 5 240 247 10.1177/1740774508091749 18559413 PMC2757268

